# The effect of aloe emodin–encapsulated nanoliposome‐mediated r‐caspase‐3 gene transfection and photodynamic therapy on human gastric cancer cells

**DOI:** 10.1002/cam4.584

**Published:** 2015-12-21

**Authors:** Kai‐Ting Li, Qin‐Qin Duan, Qing Chen, Juan‐Wen He, Si Tian, Hai‐Dan Lin, Qing Gao, Ding‐Qun Bai

**Affiliations:** ^1^ Department of RehabilitationThe First Affiliated Hospital of Chongqing Medical UniversityChongqingChina; ^2^ Department of gastroenterologyChinese Medicine Hospital of LongquanChengduChina; ^3^ Department of gastroenterologyThe First Affiliated Hospital of Chongqing Medical UniversityChongqingChina

**Keywords:** Aloe emodin, nanoliposome, photodynamic therapy, transfection

## Abstract

Gastric carcinoma (GC) has high incidence and mortality rates in China. Surgery and chemotherapy are the main treatments. Photodynamic therapy (PDT) has become a new treatment modality, appearing in recent experimental studies and clinical trials in various tumors. This study explores the combined effect of gene transfection with PDT on GC cells using aloe emodin (AE)–encapsulated nanoliposomes, which acted as gene carrier as well as one photosensitizer (PS). AE‐encapsulated nanoliposomes (nano‐AE) were prepared by reverse evaporation method. Electron microscopy and nano‐ZS90 analyzer were used to detect its morphology, size, and wavelength. Western blot was used to detect the expression of the caspase‐3 after transfection. MTT assay and flow cytometry were employed to determine the cytotoxic and apoptotic rates, respectively. Hoechst 33342 staining was adopted to detect the morphological changes in death gastric cancer cells. Cellular reactive oxygen species (ROS) contents were measured by DCFH‐DA staining. Outcomes demonstrated that the nano‐AE has good properties as gene delivery carriers as well as a PS. The group in which the recombinant plasmid of r‐caspase‐3 was transfected had higher protein expression of the caspase‐3 than controls, meanwhile the proliferation rates of the transfected cells were inhibited by the nano‐AE‐mediated PDT in an energy‐dependent manner. In addition, in the transfected cells, the death rate increased to 77.3% as assessed 12 h after PDT (6.4 J/cm^2^). Hochest 33342 staining also revealed that the death rate increased significantly in the transfected group compared with other groups. Compared to control groups, the production of ROS in nano‐AE PDT group had quadrupled in SGC‐7901 cells as early as 1 h after PDT, while it is similar to the group of nano‐AE transfection and PDT. Nano‐AE‐mediated r‐caspase‐3 gene transfection coupled with PDT could inhibit the proliferation rate and increase the apoptotic rate remarkably in human gastric cancer cells.

## Introduction

Photodynamic therapy (PDT) is a new treatment modality that has arisen within the recent 30 years. Compared with traditional therapies, such as surgery, radiotherapy, and chemotherapy, PDT has several advantages like minimal invasion, few toxic side effects, and low mutation rate 1. Owing to these factors, PDT has been increasingly used to treat tumors in experimental studies and clinical trials. PDT comprises two steps: (1) the photosensitizer (PS) selectively accumulates in tumor tissues and (2) photochemical reactions occur under light irradiation of appropriate wavelengths, and singlet reactive oxygen species (ROS) are generated in these reactions, which leads to photodamage of tumor cells, interruption of tumor blood supply, and stimulation of the immune response 1, 2. PDT has been used in the treatment of diseases such as bacterial infections, age‐related macular degeneration, and actinic keratosis 3, 4, 5, 6. However, the greatest application of PDT may be in the fight against cancer, such as esophageal cancer, gliomas, breast cancer 7, 8, 9. In the process of PDT, three major components are required: PS, a source of high‐energy visible light, and tissue oxygen. PS are thought to be a core material in PDT, and play a decisive role in the reaction.

Aloe emodin (AE) is a potential PS that has demonstrated antitumor effects 10, 11, 12. However, AE is poorly water soluble, thus oral absorption and bioavailability may be poor. Meanwhile, long‐term use of AE may lead to adverse reactions such as acute renal failure 13. These issues limit its widespread application in the field of medicine. Therefore, research efforts to improve the water solubility of AE have important significance to increase its bioavailability. Nanoliposomes with good biological distribution, good bioavailability, and low drug toxicity are thought to be the best drug carriers for solid tumor therapies 14. At the same time, nanoliposomes, as a carrier, are becoming more and more important in the process of transfecting recombinant plasmid into cells. Thus, we prepared the AE‐encapsulated nanoliposomes (nano‐AE), which carried the wish of not only improving the bioavailability of AE, but also transfecting recombinant plasmid as a carrier. Caspase‐3 is a protein enzyme that plays a critical role in apoptosis; however, it must be cleaved before it becomes active 15. The molecular r‐caspase‐3 can fold spontaneously into a 3D structure and can generate constitutive activity by rearrangement (rather than by cleavage) 16. In doing so, activation of caspase‐dependent apoptosis can be achieved.

Gastric cancer is one of the most common malignant diseases in the world and is the second leading cause of cancer deaths 17. Gastric cancer that is limited to the stomach and regional lymph nodes is potentially curable by surgical resection. However, approximately 20–40% of all patients that undergo resection without further treatment will suffer a relapse within 2 years 18. Once a relapse occurs, no curative treatment is available and the median survival is approximately 6 months 18. Thus, it is important to find a better treatment. PDT has been used in many fields because of its special advantages. Accordingly, in this study, we investigated the effect of transfection of r‐caspase‐3 with nano‐AE and PDT on human gastric cancer cells.

## Materials and Methods

### Reagents, DNA, and cell line

The plasmid pcDNA 3.1(−)/r‐caspase‐3 was constructed by the investigators 19. AE was purchased from Jiangxi Tiangong Technology (Jiangxi, China), and dimethylsulfoxide (DMSO), l‐*α*‐phosphatidylcholine, cholesterol, 3‐2,5‐diphenyl‐tetrazolium bromide (MTT), and Hochest33342 were purchased from Sigma‐Aldrich Co. (St. Louis, MO). Caspase‐3 antibodies were obtained from Cell Signaling Technology (Danvers, MA) and *β*‐actin antibodies and the corresponding secondary antibodies were purchased from Santa Cruz Biotechnology (Santa Cruz, CA). RPMI 1640 medium was purchased from Hyclone (Logan, UT) and fetal bovine serum (FBS) was purchased from Biological Industries (Kibbutz Beit Haemek, Israel). The light‐emitting diode (LED) light was purchased from Chongqingjingyu Laser Biological Company (Chongqing, China). Human gastric cancer cell line (SGC‐7901) was purchased from the Shanghai Institute of Biochemistry and Cell Biology, Chinese Academy of Sciences, China.

### Nano‐AE preparation and characterization

The AE stock solution (100 mmol/L) was prepared in DMSO, filtered with a 0.2‐*μ*m polytetrafluoroethylene syringe filter to remove any insoluble compounds, and stored in the dark at −20°C. An l‐*α‐*phosphatidylcholine/cholesterol (75 mg:25 mg) mixture containing the AE (1 mg) dissolved in 1 mL anhydrous ethanol was added to a round bottom flask with 10 mL chloroform. The mixture was evaporated to dryness using a dry tetrahydrofuran solution at 40°C with vacuum relief in order to form a uniformly thin film. Next, 10 mL chloroform and 3 mL phosphate‐buffered saline (PBS) were added to the round bottom flask and shocked and ultrasonified (5 min, 37°C) to enable formation of the stable water‐in‐oil (W/O) phase. The mixed suspension of liposomes was prepared by stable constriction using a particle vacuum rotator (RE‐52AA Vacuum rotary evaporation apparatus [Shanghai Rong Biochemical Instrument Plant, Shanghai, China]) at 40°C, and then 7 mL of PBS was added to wash continually at atmospheric pressure until the suspension liposomes harvested. Finally, the shape of the nanoliposomes was observed by transmission electron microscope. Briefly, a drop of nanoliposomes was added onto the bronze grid and the excess liquid was absorbed from the edge with filter paper. Next, the liposomes were stained and dried. The shape of the nanoliposomes observed by the transmission electron microscope (H‐7500; Hitachi, Tokyo, Japan) appeared to be neat and spherical.

### Measurements of nano‐AE size and wavelength

The sample of nanoliposomes (0.5 mL) was diluted 10 times. The size was detected by a nano‐ZS90 analyzer (Malvern, Worcestershire, UK). Nanoliposome (2 mL, prepared 2000r/min) of membrane was ruptured by 10% Trition‐X‐100 (1 mL) and scanned by full wavelength ultraviolet spectrophotometer.

### Cell culture

The gastric cancer cells SGC‐7901 were cultured in RPMI 1640 medium supplemented with 10% FBS. The cells were incubated at 37°C in a humidified atmosphere containing 5% CO_2_ for 1–2 days to attach and spread. The cells in the growth period were used for the next experimentation.

### Transfection biology

The gene delivery efficiency of the nano‐AE was investigated by western blot. The SGC‐7901 cells were plated at a density of 3 × 10^5^ cells/well in six‐well plates and incubated with an atmosphere of 5% CO_2_ at 37°C for 24 h. The medium was then replaced with the complete RPMI 1640 (1 mL) containing various amounts of the nano‐AE (2–24 *μ*L) and 2 *μ*g of pDNA (1/1 to 12/1 v/w ratio). After 6 h of transfection of the SGC‐7901 cells, the medium was replaced with culture medium (2 mL) with FBS (10%) and then the cells were cultured for 72 h. Cells were collected and washed twice with PBS, and then total proteins were extracted in lysis buffer (Kaiji Bio Co., Nanjing, China). The resulting solution was collected and subjected to polyacrylamide gel electrophoresis (SDS‐PAGE, 10% resolving gel). The protein bands were transferred to a nitrocellulose membrane using the wet transfer method. The membrane was blocked with 5% nonfat dry milk in TBST for 1 h at room temperature. After washing, the membrane was incubated overnight with a primary antibody at 4°C. The membrane was then washed and incubated with a secondary antibody for 1 h. The immunoreactive bands of protein were developed using an enhanced chemiluminescent substrate (Kaiji Bio Co.) and exposed using the G:BOX iChemi XR gel documentation system (Syngene, Cambridge, UK). The gray value of each lane was analyzed using Image J software (NIH, Bathesda, MD). Three independent experiments were performed and the representative results were shown.

### MTT reduction assay

The SGC‐7901 cells (5 × 10^3^ cells/plate) growing in a 96‐well plate were cultured for 24 h. Cells were divided into four groups—A: without nano‐AE and no pDNA, treated as blank control; B: pDNA group; C: nano‐AE group; D: nano‐AE transfection group. Group A was cultured with fresh culture RPMI 1640 medium (100 *μ*L) free of FBS. Group C was incubated with RPMI 1640 medium (100 *μ*L) free of FBS but with nano‐AE (0.6 *μ*L). Groups B and D were incubated with complete RPMI 1640 (100 *μ*L) containing the nano‐AE (0.6 *μ*L) and pDNA (0.2 *μ*g) for 6 h. Then the mediums were replaced by culture medium (100 *μ*L) with FBS (10%) and the cells were then cultured for 72 h. Cells were exposed (except for the dark controls) to UV light from LED at a wavelength of 430 nm and an energy density of 40 mw/cm^2^ for 0, 20, 40, 80, 160, 320, or 640 sec. Final energy density of 0, 0.8, 1.6, 3.2, 6.4, 12.8, and 25.6 J/cm^2^ was achieved, respectively. Cells were cultured for 20 h in the dark. Next, the cells were incubated in medium with MTT (5 *μ*g/mL, 20 *μ*L) for 4 h, and then 150 *μ*L DMSO was added to each well and the cells were shocked at room temperature for 10 min. The optical density (OD) was measured in triplicate using an iEMS Analyzer at wavelength of 570 nm. The percentage of cytotoxicity was calculated using the following equation: Cell viability rate (%) = OD treatment group/OD control group × 100%. Experiments were repeated five times.

### Flow cytometry

SGC‐7901 cells (4.5 × 10^5^ cells/plate) growing in six‐well plates were cultured for 24 h. Cells were divided into eight groups—A: without nano‐AE, pDNA and no light, treated as blank control; B: pDNA group; C: nano‐AE group; D: nano‐AE transfection group; E: pure light group; F: pDNA and light group; G: nano‐AE PDT group; H: nano‐AE transfection and PDT group. Groups A and E were cultured with fresh culture RPMI 1640 medium (1 mL) free of FBS. Groups C and G were incubated with RPMI 1640 medium (1 mL) free of FBS with liposomes (6 *μ*L). Groups B, D, F, and H were incubated with the mixture (1 mL) containing the nano‐AE (6 *μ*L) and pDNA (2 *μ*g). All groups were incubated for 6 h. The medium was replaced with culture medium (2 mL) with FBS (10%) and was cultured for 72 h. Cells were exposed (except for the dark controls) to UV light from the LED at a wavelength of 430 nm and an energy density of 40 mw/cm^2^ for 160 sec (the final energy density of 6.4 J/cm^2^). Cells were then cultured for 12 h in the dark. All cells were harvested for Annexin V/PI double staining and measured using a BD FACScalibur flow cytometry system (BD Bioscience, Franklin lakes, NJ).

### Hoechst nuclear staining

SGC‐7901 cells (1 × 10^4^ cells/plate) growing on 24‐well plates were cultured for 24 h. Cells were divided into eight groups. The groups dividing and the incubation methods were the same as we did in flow cytometry detection. After 12 h of PDT, the cells were washed three times with PBS and were stained by Hoechst 33342 (2 *μ*g/mL) for 20 min, and observed with fluorescent microscopy under ultraviolet illumination.

### ROS detection

Cellular ROS contents were measured by flow cytometry. Briefly, SGC‐7901 cells (5 × 10^5^/well) were plated on 60‐mm dishes and were allowed to grow overnight. Cells were divided into six groups—A: without nano‐AE, pDNA and no light, treated as blank control; B: nano‐AE group; C: pure light group; D: pDNA and light group; E: nano‐AE PDT group; F: nano‐AE transfection and PDT group. Then the incubation methods of every group were the same as we depicted in flow cytometry detection. Cells were then cultured for 1 h in the dark. All cells were harvested for DCFH‐DA (Invitrogen, Paisley, UK) staining and measured using BD FACScalibur flow cytometry system (BD Bioscience).

### Statistical analysis

Experimental data are shown as mean ± standard deviation using SPSS 17.0 (IBM, New York, NY) for each data to test for normality and homogeneity test of variance. The comparison between multiple sets of data was performed using a single factor analysis of variance and a two‐factor analysis of variance, and the comparison of the two groups was performed using LSD analysis. *P *<* *0.05 was considered statistically significant.

## Results

### Characteristics and morphology of nanoliposomes

**Figure 1:**
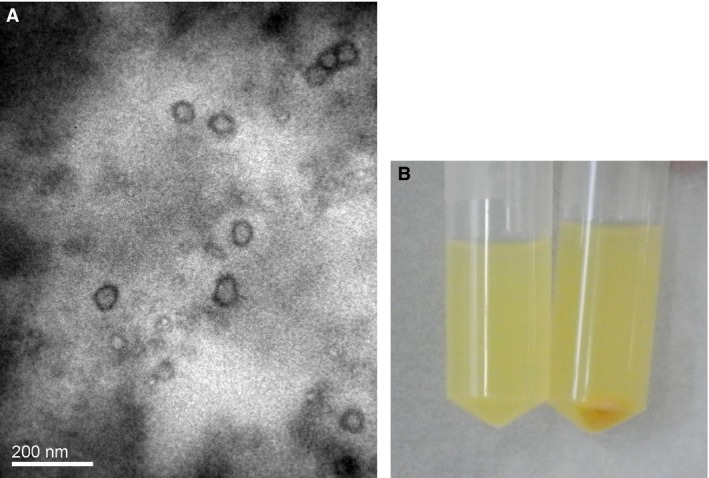
(A) Morphology of nano‐AE (aloe emodin–encapsulated nanoliposomes) under transmission electron microscope (×10,000). (B) Nano‐AE liquid suspension and precipitation particles.

Nano‐AE had spherical shapes and embedded in the double walls of the liposomes observed by transmission electron microscope, as shown in Figure 1A. Nano‐AE suspension liquid and centrifugal precipitation particles are shown in Figure 1B.

### Nanoliposomes wavelengths

**Figure 2:**
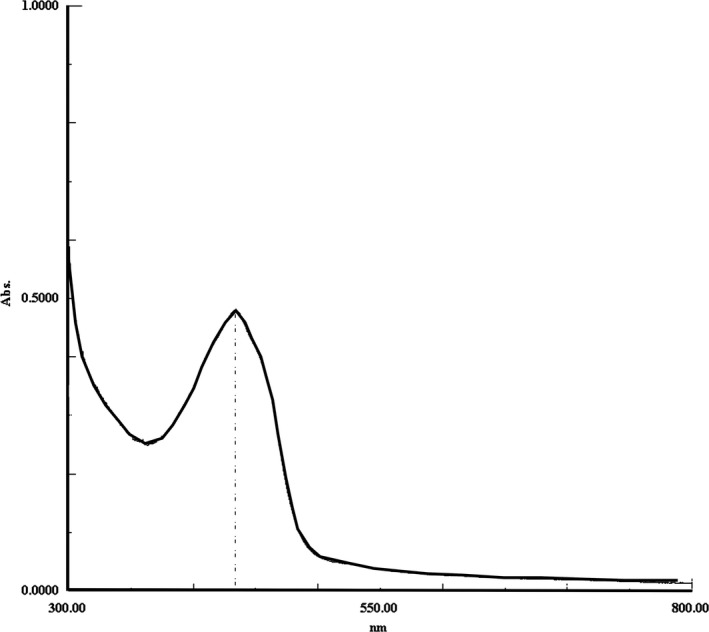
Absorption wavelengths of nano‐AE (aloe emodin–encapsulated nanoliposomes).

Wavelength distribution of the PBS aqueous solution of nano‐AE under the full wavelength scanner is shown in Figure 2. The data showed that the absorption wavelength of nano‐AE was nearly 430 nm, which is the nature absorption wavelength of AE.

### Particle size distribution of nanoliposomes

**Figure 3:**
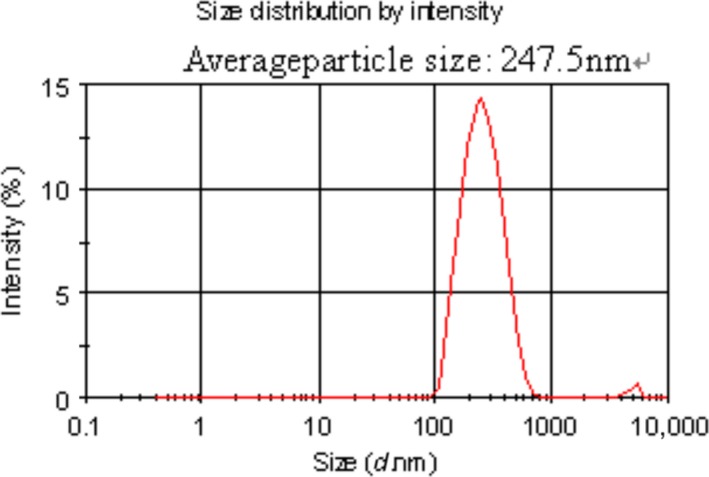
The particle size distribution of nano‐AE (aloe emodin–encapsulated nanoliposomes).

Particle size distribution of nano‐AE PBS aqueous solution was shown in Figure 3 detected using nano‐ZS90 (Malvern). And according to the results, the size nano‐AE was distributed between 100 and 800 nm, and the average particle size was 247.5 nm, which is available.

### Western blot to detect the transfection biology

**Figure 4:**
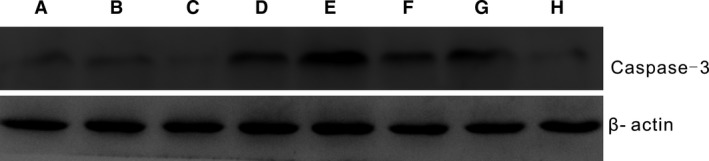
Caspase‐3 protein expression after r‐caspase‐3 genes transfection of different proportions of nano‐AE (aloe emodin–encapsulated nanoliposomes) and pDNA; A: blank control group; B: nano‐AE group; C: pDNA group; D: transfection group 1/1 (2 *μ*L: 2 *μ*g); E: transfection group 3/1 (6 *μ*L: 2 *μ*g); F: transfection group 6/1 (12 *μ*L: 2 *μ*g); G: transfection group 9/1 (18 *μ*L: 2 *μ*g); H: transfection group 12/1 (24 *μ*L: 2 *μ*g).

After the recombinant plasmid of r‐caspase‐3 gene was transfected under different proportions of nano‐AE and pDNA mixture, the expression of caspase‐3 was observed by western blot. Results showed that the expression of caspase‐3 increased in varying lipid‐to‐pDNA charge ratio groups, and the highest transfection efficiency was obtained at 3/1 (6 *μ*L: 2 *μ*g) ratio, as shown in Figure 4.

### The inhibitory effect of nano‐AE‐mediated r‐caspase‐3 gene transfection and PDT on human gastric cancer cells

**Figure 5:**
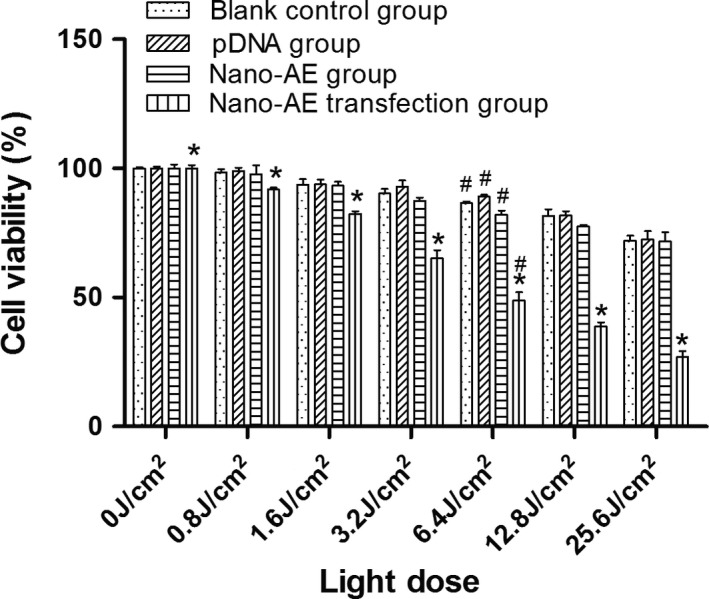
The inhibitory effect of nano‐AE (aloe emodin–encapsulated nanoliposomes)‐mediated r‐caspase‐3 gene transfection and photodynamic therapy (PDT) on human gastric cancer cells. *Nano‐AE transfection PDT group versus blank control group, pDNA group, and nano‐AE group, *P* < 0.01; #mutual comparison in the four groups, *P* < 0.05. Values are mean ± SD of three independent determinations.

The results of the inhibitory effect of nano‐AE‐mediated PDT after r‐caspase‐3 gene transfection at varied energy densities are shown in Figure 5. It was observed that the greater the light energy, the lower the survival rate of the cells. The differences in inhibition rate between the different energies and the control group (no light) were statistically significant (*P < 0.01). Under the action of different light energies, the differences in inhibition rate of nano‐AE transfection and PDT group were statistically significant when compared with the blank control group, the pDNA group, and the nano‐AE group (#*P *< 0.01). Nevertheless, there was no obvious inhibition of cellular proliferation noted in the groups without light irradiation (*P* > 0.05). Besides, nano‐AE mediated r‐caspase‐3 gene transfection and PDT on gastric cancer cells with an IC_50_ of 6.4 J/cm^2^, as shown in Figure 5. Therefore, the related experiment below used 6.4 J/cm^2^.

### Death of nano‐AE mediated r‐caspase‐3 gene transfection and PDT on gastric cancer cells

**Figure 6:**
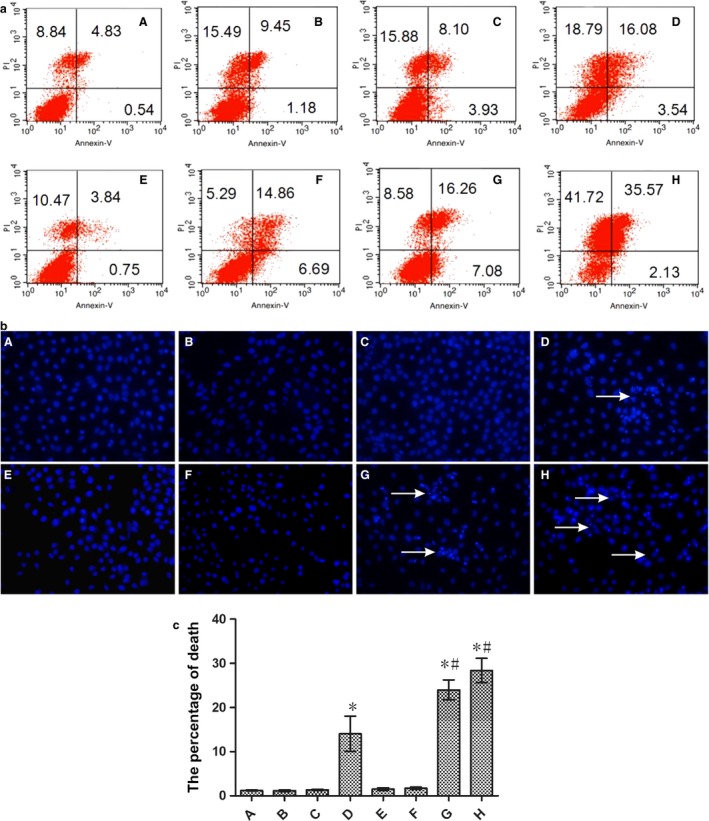
(a) Apoptosis of nano‐AE (aloe emodin–encapsulated nanoliposomes)‐mediated r‐caspase‐3 gene transfection and photodynamic therapy (PDT) on gastric cancer cells (6.4 J/cm^2^). (b) Morphological changes in the cell nuclei undergoing nano‐AE‐mediated r‐caspase‐3 gene transfection and PDT on gastric cancer cells (6.4 J/cm^2^). (c) The percentage of death quantified by the Hoechst stain. A: blank control; B: pDNA group; C: nano‐AE group; D: nano‐AE transfection group; E: pure light group; F: pDNA and light group; G: nano‐AE PDT group; H: nano‐AE transfection and PDT group. *P < 0.05, Group D/G/H versus Group A. #*P* < 0.05, Group G/H versus Group D. Values are mean ± SD of three independent determinations.

The apoptosis rate of nano‐AE‐mediated r‐caspase‐3 gene transfection and PDT (6.4 J/cm^2^) in gastric cancer cells was assessed with Annexin V/PI double staining. Results showed that the apoptosis rate of the H group (nano‐AE transfection and PDT group) was significantly higher than that of other groups (*P* < 0.01). The apoptosis rate of gastric cancer cells in the H group was higher than that in the D and G groups (*P* < 0.01), as shown in Figure 6a. However, when focusing on the population of cells that were undergoing apoptosis, the percentage of this population did not increase correspondingly in panel H in Figure 6b. Therefore, it appears that another form of cell death was taking place and contributing to the increase of overall cell death events.

### Morphological changes in the cell nuclei undergoing nano‐AE‐mediated r‐caspase‐3 gene transfection and PDT

Cell apoptosis of nano‐AE‐mediated r‐caspase‐3 gene transfection and PDT on gastric cancer cells was also assessed by Hoechst 33342. The changes in the cell nuclei were observed under the fluorescence microscope. With Hoechst staining, it was observed that nuclear chromatin density increased, as indicated by a bright blue color and distinct features of condensation, coagulation, and fragmentation of the nuclear chromatin, as well as typical apoptotic bodies in the nano‐AE transfection and PDT groups, as shown in Figure 6b. At the same time, we found that in groups of pDNA transfection and nano‐AE PDT, the Hoechst‐positive cells increased compared with the controls. The percentage of the death cells possessed 28.4 ± 2.8, 24.0 ± 2.2, 14.0 ± 4.0 in nano‐AE transfection and PDT group, nano‐AE PDT group, and pDNA transfection group, respectively, while in the other control groups, the percentage was very low (Fig. 6c).

### Nano‐AE‐mediated PDT triggered ROS production

**Figure 7:**
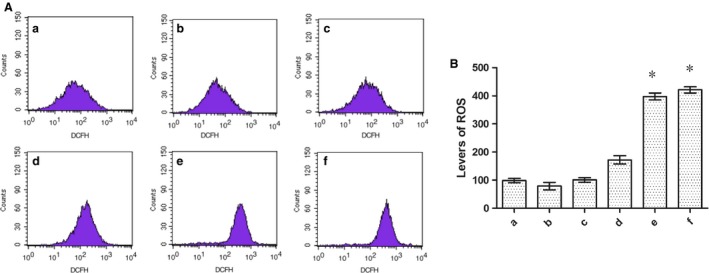
(A) ROS detected by DCFH‐DA staining. (B) Levels of ROS. (a) blank control; (b) nano‐AE (aloe emodin–encapsulated nanoliposomes) group; (c) pure light group; (d) pDNA and light group; (e): nano‐AE photodynamic therapy (PDT) group; (f): nano‐AE transfection and PDT group. *P < 0.05, Group e/f versus Group a. Values are mean ± SD of three independent determinations.

It has been recognized that ROS production in cancer cells triggered by PDT is one of the main mechanisms. ROS can induce damages to mitochondria, endoplasmic reticulum, and other organelle, thereby inducing apoptosis 17. DCFH‐DA staining showed that there had more ROS in the nano‐AE PDT group as well as nano‐AE transfection coupled with PDT group compared with other groups (*P* < 0.05). Compared to control groups, the production of ROS had quadrupled in SGC‐7901 cells as early as 1 h after PDT (Fig. 7). The data suggested that nano‐AE PDT generated ROS and lead to apoptosis.

## Discussion

Nanoliposome is an effective drug delivery carrier for gene therapy and PDT 19. In the present study, we successfully prepared nano‐AE with the mean particle size 247.5 nm. In addition, nanoliposomes did not appear to change the nature of the drug; the PBS solution of nano‐AEs had the same maximum absorption wavelength as AE. In this study, we chose SGC‐7901 cells as the tumor model cells and applied the nano‐AE as a delivery carrier for plasmid r‐caspase‐3 transfection as well as one nano‐PS for PDT.

In our study, we constructed a eukaryotic expression plasmid of pcDNA3.1(–)/r‐caspase‐3, and nano‐AE could effectively combine the plasmid and transfected into the gastric cancer cells. As shown in Figure 4, different nano‐AE/r‐caspase‐3 ratios were prepared and western blot was used to check caspase‐3 protein expression. Results showed that the expression of the caspase‐3 protein significantly increased in the cells treated by nano‐AE‐mediated gene transfection, suggesting that nano‐AE could significantly enhance the transfection efficiency of r‐caspase‐3 into the SGC‐7901 cells. Besides, results showed that the highest expression of caspase‐3 occurred at a ratio of 3:1 (6 *μ*L:2 *μ*g). Therefore, we chose the ratio in the present study.

After r‐caspase‐3 transfection, the transfected cells were irradiated by a LED light source of 430 nm to explore the combined effect of gene transfection and photodynamic treatment using nano‐AE. Light‐dependent photocytotoxicity of the nano‐PS in the SGC‐7901 cells was observed in the MTT assay. Under the same conditions of incubation, the photosensitizing activity of the nano‐PS increased with the light dose. The results indicated that nano‐AE was an effective PS and its photodynamic efficacy depended on the light doses.

The recombinant form of r‐caspase‐3 induced apoptosis, as shown in Figure 6a, and the apoptosis rate of these gastric cancer cells was significantly higher than that in blank control group, pure light group, pDNA group, and nano‐AE group (*P* < 0.01).

Photodynamic therapy as a new treatment has been widely used in the treatment of tumors. In recent years, research regarding AE as a PS‐mediated PDT on tumors has been relatively rare because AE was a nonsoluble PS, which was inconvenient for experimental studies and clinical research. We demonstrated that nanoliposomes can alter the solubility of AE in water, improve the bioavailability, and do not affect its properties, which brought a new hope for future studies. The apoptotic mechanisms behind PDT on tumor cells may be any of the following: (1) directly kill tumor cells, (2) destruct blood vessels of tumors, and (3) induce immune response 1. However, the photodynamic‐mediated products of molecular oxygen and ROS are the main mechanisms of cell death 8. When the light energy density is increased to a certain extent, apoptosis translates into nonprogrammed death or necrosis 1. Just as shown in our study, nano‐AE acted well as a PS, and induced the production of ROS after irradiation. Inhibitory effect of nano‐AE‐mediated PDT after r‐caspase‐3 gene transfection on gastric cancer cells appeared in an energy‐dependent manner. Moreover, the greater the light energy, the lower the cell survival rate was, as shown in Figure 5. Besides, the apoptosis rate of gastric cancer cells increased from 5.37% to 37.7%, the necrosis rate increased from 8.84% to 41.72%, when compared with control groups, just as shown in Figure 6a. These results suggested that nano‐AE‐mediated r‐caspase‐3 gene transfection and PDT had a significant killing effect on gastric cancer cells. The necrosis rate in the nano‐AE transfection and PDT group was high, thus, under light intensity, both necrosis and apoptosis may result in the death of gastric cancer cells. The specific reason and mechanisms behind this require further research.

## Conclusions

In this study, we demonstrated that nano‐AE can be produced by reverse evaporation, with spherical morphology, and of which had a maximum absorption wavelength at 430 nm. Besides, nano‐AE maintained its photosensitive property. The killing effect of nano‐AE‐mediated r‐caspase‐3 gene transfection and PDT on the gastric cancer cells was clear. Inducing the apoptosis and necrosis of gastric cancer cells may be the main mechanism, but further studies are warranted to identify concrete pathways.

## Conflict of Interest

None declared.
